# Case Report: Multi-targeted therapy in the treatment of severe toxic epidermal necrolysis

**DOI:** 10.3389/fped.2024.1460579

**Published:** 2024-11-22

**Authors:** Elaine Yi Lee Kwong, Manson Chon In Kuok, King Fai Lam, Winnie Kwai Yu Chan

**Affiliations:** Department of Paediatrics, Queen Elizabeth Hospital, Kowloon, Hong Kong SAR, China

**Keywords:** toxic epidermal necrolysis, Stevens–Johnson syndrome, trimethoprim–sulfamethoxazole, etanercept, cyclosporin A, ocular involvement

## Abstract

We reported a 10-year-old child who suffered from severe toxic epidermal necrolysis triggered by trimethoprim–sulfamethoxazole and managed successfully with multi-targeted therapy. He was jointly managed by a paediatric intensivist, a dermatologist, an otolaryngologist, a urologist, a wound nurse, a pain management specialist, a dietitian, and a clinical psychologist. Systemic intravenous immunoglobulin and pulsed-dose methylprednisolone were initiated after admission. Oral cyclosporin A was added in the early stage of the disease in view of severe ocular involvement with progressive inflammation of bilateral upper and lower eyelids, the presence of pseudomembrane, diffuse conjunctival injection, and progression of central epithelial defects in bilateral eyes. He underwent amniotic membrane transplantation. Subcutaneous injection of etanercept was added on the treatment to allow rapid tapering of steroids. Finally, the disease progression was halted with re-epithelisation on day 13. He experienced no side effects from the multi-targeted therapy and recovered well without clinical sequelae.

## Case presentation

**Figure 1:**
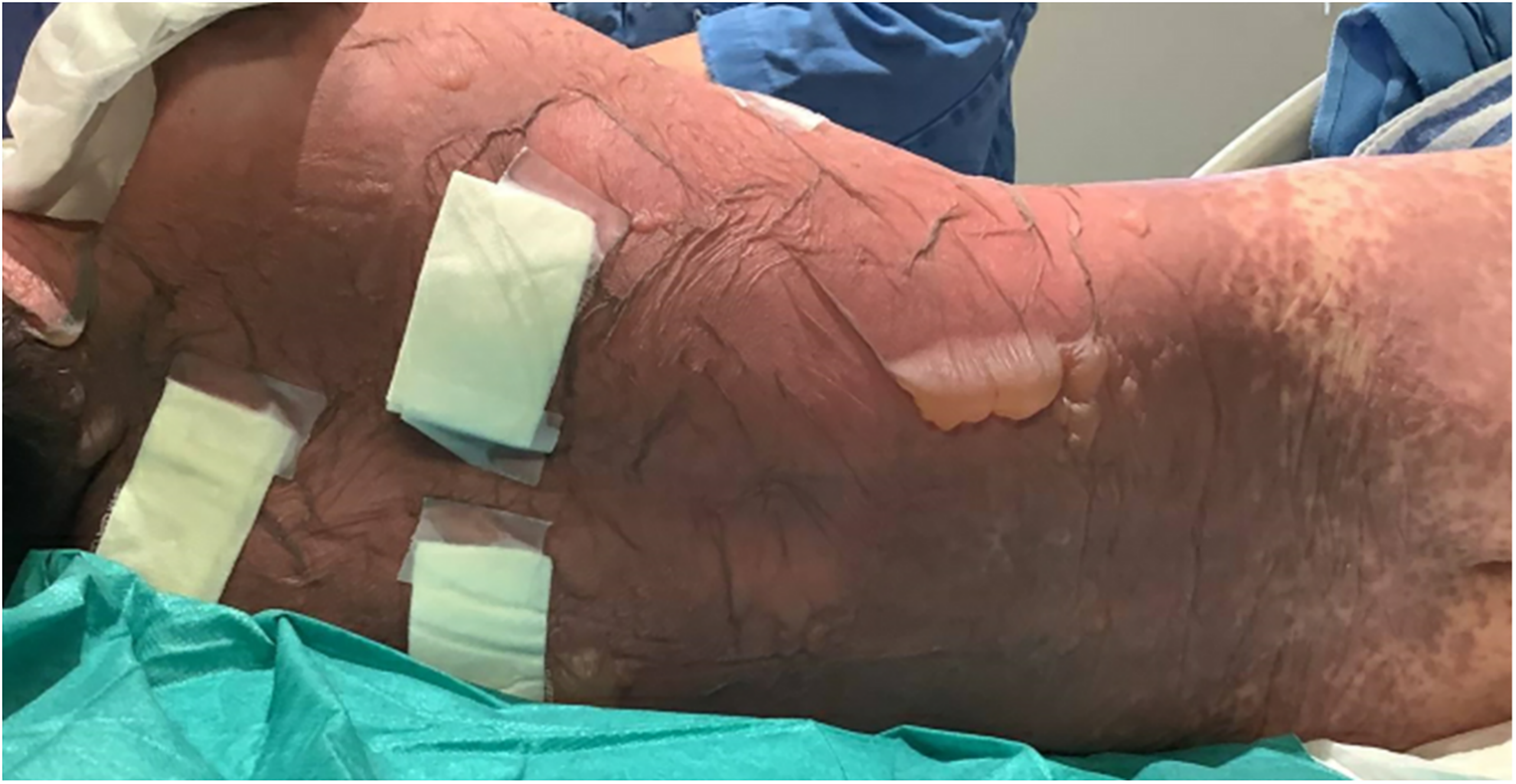
Extensive bullae formation over the back of the patient, which ruptured easily with positive Nikolsky's sign and diffuse detachable skin.

**Figure 2:**
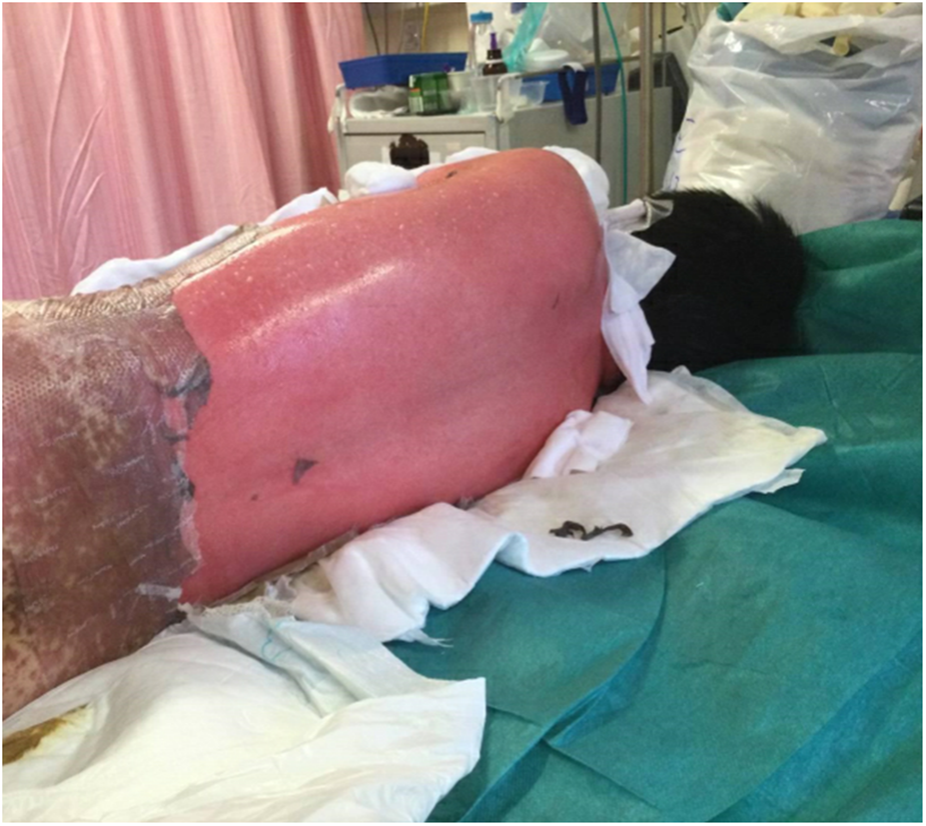
The back of patient showing the denuded area due to epidermal necrolysis.

**Figure 3:**
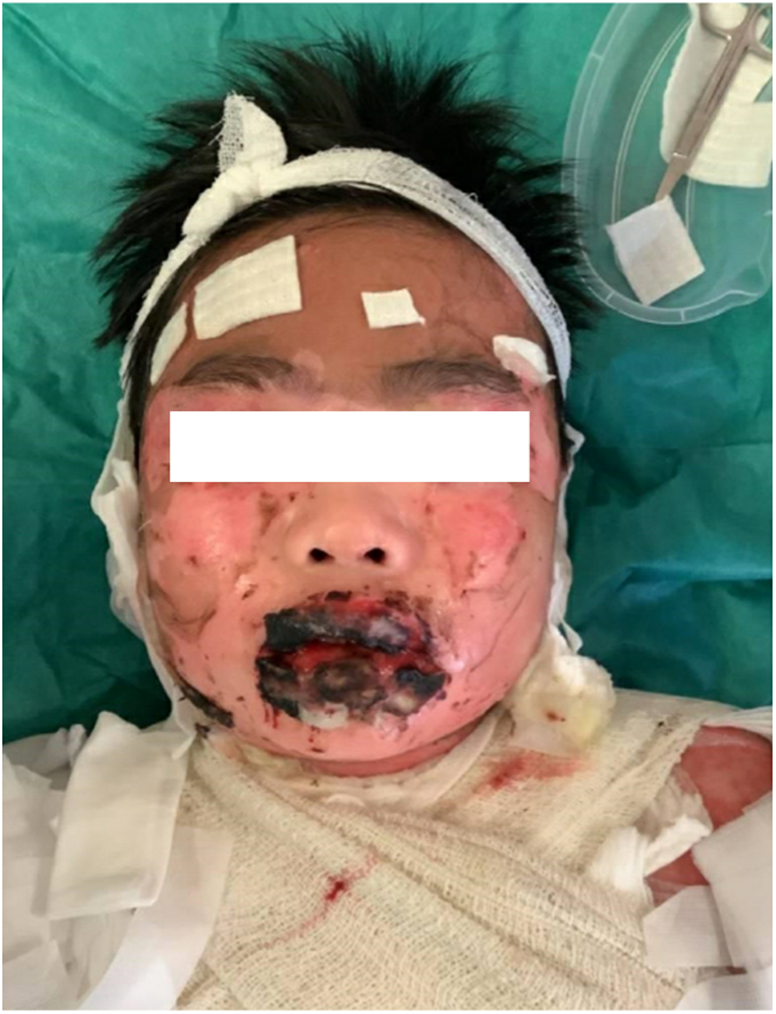
The blistering lesions on the face with epidermis sloughed off and haemorrhagic crusting of the lips.

A 10-year-old previously well Chinese boy presented to an orthopaedic doctor with fever, left leg redness, and pain. He was treated for left leg cellulitis with intravenous amoxicillin–clavulanate and oral trimethoprim–sulfamethoxazole. He responded to treatment initially but developed fever and headache 10 days after taking the antibiotics. Over the next 4 days, he developed high-grade fever and diffuse erythematous rashes over his face, neck, and body. The lesions started as painful erythematous macules and then progressed to blisters followed by diffuse bullae over his lips, ear pinnae, trunk, bilateral extremities, and genitalia within a few hours (Figures 1–3). His drug history revealed that he had taken oral amoxicillin–clavulanate 3 years ago which he tolerated well without experiencing adverse drug reactions.

At presentation to the paediatric intensive care unit, the patient was febrile with a temperature of 40.7°C, tachycardic with a pulse rate of 159/min, and had a blood pressure of 116/72 mmHg. Physical examination was remarkable for diffuse erythema, bullae, and extensive epidermal detachment involving around 50% body surface area (BSA). Nikolsky's sign was positive. In addition, he had conjunctival injection, facial oedema, haemorrhagic crusting of lips, and erosion over the penile shaft. He had no respiratory distress. The chest, cardiovascular, and abdominal examination was normal. A clinical diagnosis of toxic epidermal necrolysis (TEN) was made.

The complete blood count showed leukopenia (2.7 × 10^9^/L), lymphopenia (0.3 × 10^9^/L), haemoglobin of 10.2 g/L, and normal platelet count of 185 × 10^9^/L. He had disseminated intravascular coagulation with a prolonged prothrombin time (PT) of 15 s (reference 10.7–13.1 s), activated partial thromboplastin time (APTT) of 34.7 s (reference 25.8–33.8 s), international normalised ratio (INR) of 1.36, fibrinogen of 2.4 g/L (reference 1.5–3.6 g/L), and elevated D-dimer at 1,229 ng/ml FEU (reference <500 ng/mL FEU). Inflammatory markers were significantly elevated. Procalcitonin was elevated at 4.74 ng/ml (reference <2 ng/ml). C-reactive protein (CRP) was 89 mg/L (reference <5 mg/L). Erythrocyte sedimentation rate (ESR) was 98 mm/h (<17 mm/h). Lactate dehydrogenase (LDH) was 651 IU/L (reference 120–325 IU/L). There was a reversal of albumin to globulin ratio. Albumin was 26 g/L (reference interval 37–47 g/L). Globulin was 51 g/L (reference range 24–37 g/L). He had hyponatraemia (sodium 132 mmol/L), hypokalaemia (potassium 3.4 mmol/L), hypocalcaemia (calcium 1.98 mmol/L) and hypophosphatemia (phosphate 0.7 mmol/L). Serum urea was 2.7 mmol/L (reference 1.8–6.4 mmol/L). Bicarbonate was 19 mmol/L (reference 22–29 mmol/L). Glucose was 7.4 mmol/L. Transaminases were mildly elevated with alanine aminotransferase (ALT) at 59 IU/L (reference <44 IU/L) and aspartate aminotransferase (AST) at 103 IU/L (reference <48 IU/L). The lip swab yielded methicillin-resistant *Staphylococcus aureus* (MRSA). The anal swab yielded extended-spectrum beta-lactamases (ESBL)–producing *Escherichia coli* and *Proteus mirabilis*. The swabs of lesional skin and mucous membrane were otherwise negative for herpes simplex virus (HSV), enterovirus, respiratory viruses, and mycoplasma. The serology for mycoplasma, Epstein–Barr virus (EBV), cytomegalovirus (CMV), and Herpes human virus 6 (HHV6) were negative. Blood culture yielded no growth. Chest x-ray showed no pneumonic changes. Autoimmune markers including antinuclear antibodies (ANA), anti-double-stranded DNA (anti-dsDNA), and anti-extractable nuclear antigens (anti-ENA) were negative.

**Figure 4:**
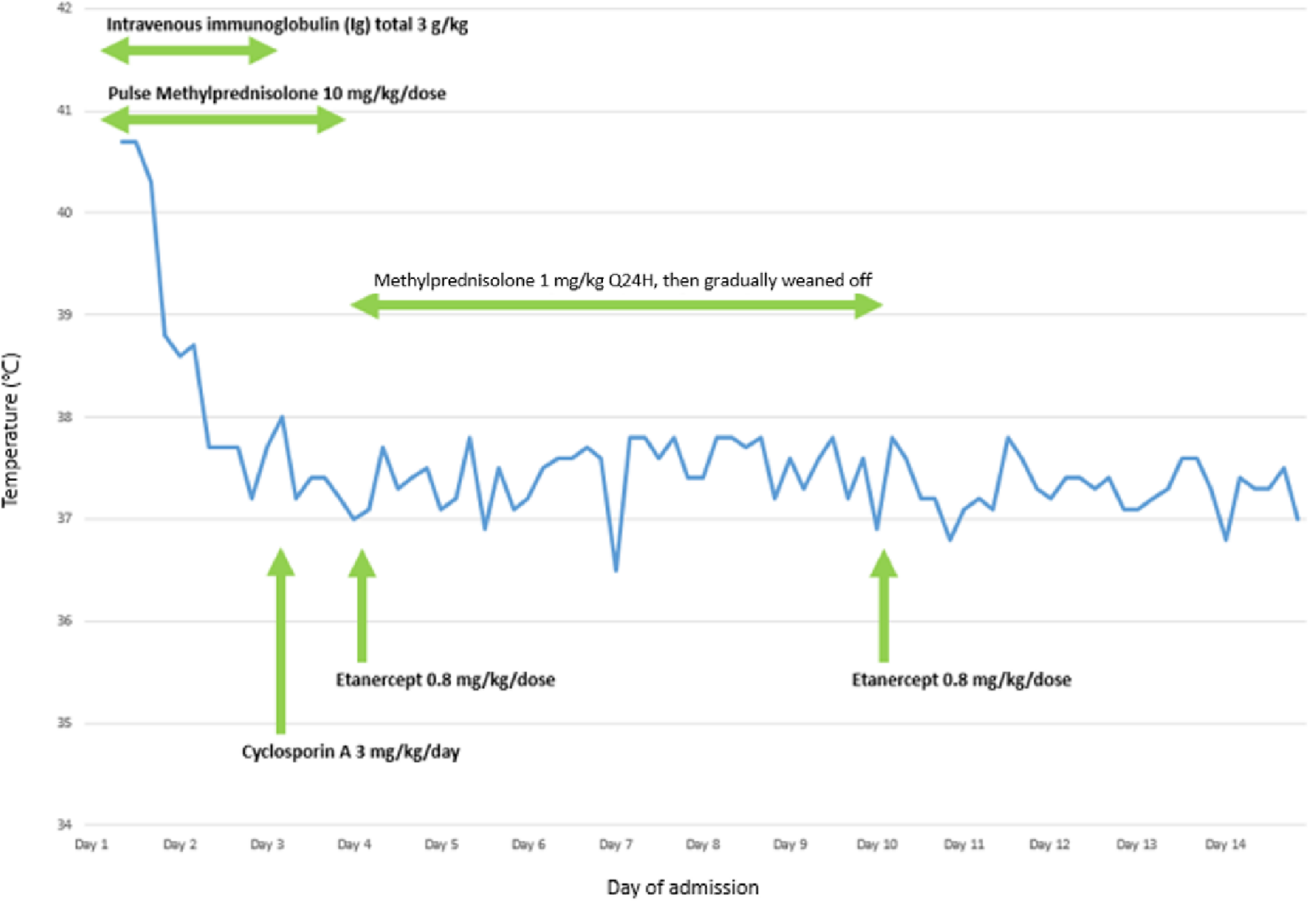
A graph illustrating the relationship between administration of different pharmacological agents and temperature of the patient during the first 14 days of hospitalisation for the treatment of TEN.

Systemic intravenous immunoglobulin (IVIG) at 1 g/kg and pulsed-dose methylprednisolone of 10 mg/kg/dose were initiated after admission and given for 3 days. Oral cyclosporin A at 3 mg/kg/day was added on day 3 of hospitalisation in view of severe ocular involvement with progressive inflammation of bilateral upper and lower eyelids, presence of pseudomembrane, diffuse conjunctival injection, and progression of central epithelial defects in bilateral eyes. He underwent amniotic membrane transplantation on day 4 of hospitalisation. Subcutaneous injection of etanercept 25 mg was administered on days 4 and 10. Finally, the disease progression was halted with re-epithelisation on day 13. Corticosteroids were gradually tapered over 1 week and cyclosporin A was given for 3 weeks in total (Figure 4).

He was jointly managed by the intensivist, dermatologist, ophthalmologist, otolaryngologist, urologist, immunologist, wound nurse, pain management specialist, dietitian, and clinical psychologist. He was given parenteral nutrition in view of possible gastrointestinal mucosal involvement, hyperhydration at 2 L/m^2^/day to cover insensible water loss, and empirical intravenous clindamycin to cover possible secondary bacterial infection. The antibiotic was subsequently stepped up to vancomycin and meropenem to target MRSA, ESBL-producing *E. coli*, and *P. mirabilis* on his lip and anal swab. Oral nystatin was prescribed as a fungal prophylaxis. The ear, nose, and throat evaluation showed evidence of erosions over the lingual surface of epiglottis and vallecula. Patency of the urinary tract was maintained by an indwelling urinary catheter, and the urethral mucosal lesion was managed with potassium permanganate. The combination of the medical therapy, dermatological and wound management with minimisation of shear forces, use of emollients to erosions, non-stick dressings, and potent topical corticosteroid ointments proved beneficial and the mucocutaneous lesions showed gradual improvement. He was finally discharged after 32 days of hospitalisation. At the last follow-up at 7 weeks from the disease onset, his skin lesions recovered well with dyspigmentation and he had no ocular sequelae so far.

## Discussion

Stevens–Johnson syndrome (SJS) and TEN are life threatening, typically drug-induced, blistering mucocutaneous diseases caused by type IV delayed-type hypersensitivity reactions. Traditionally, SJS involves <10% BSA and TEN involves >30% BSA. Overlap SJS/TEN accounts for cases with 10%–30% BSA involvement. An updated paediatric classification has been developed that defines the three entities as one disease on a spectrum, and it is called drug-induced epidermal necrolysis (DEN). Paediatric DEN is rare and affects around 7 per 100,000 patients according to data from the United States (1). The incidence of TEN is 0.4 per million children per year and the average age is 9.3 ± 0.7 years (2).

Several factors favour trimethoprim–sulfamethoxazole as the cause of TEN in our patient. Firstly, the reaction followed a reasonable temporal sequence after the drug administration. It may typically take 3–5 days for sensitisation to occur after exposure to a certain drug and 8–10 days for symptoms to develop. Our patient developed prodromal symptoms followed by mucocutaneous lesions 10 days after exposure to trimethoprim–sulfamethoxazole. Secondly, trimethoprim–sulfamethoxazole is a well-established cause of TEN in the literature. It is the most frequent cause of medication-induced SJS/TEN according to a multicentre retrospective study of 377 adult patients from the United States (3). Thirdly, the mucocutaneous lesions improved by withdrawal of the drug. Finally, the patient had no previous adverse reaction to another prescribed drug, i.e., amoxicillin–clavulanate. Thus amoxicillin–clavulanate was deemed less likely to be the culprit drug. The Adverse Drug Reaction (ADR) Probability Scale, also known as the Naranjo Scale, which was developed by Naranjo and coworkers from the University of Toronto in 1991 to assess causality for all adverse drug reactions, reveals a score of 6 suggesting that trimethoprim–sulfamethoxazole is a probable drug causing TEN (4). Trimethoprim–sulfamethoxazole is considered a very probable cause of TEN by the more specific Algorithm of Drug Causality for Epidermal Necrolysis (ALDEN) with a score of 6 (5).

The pathophysiology of TEN is believed to involve immune-mediated reactions that result in prominent keratinocyte apoptosis with epidermal necrosis and dermal–epidermal separation. Through interaction with drug antigens and the human leukocyte antigen (HLA), CD8 cytotoxic T cells and natural killer cells are induced to cause keratinocyte death. Fas-Fas ligand (FasL) interaction, perforin/granzyme B, and granulysin are well known to implicate as mediators of apoptosis (6). Therefore, various immunomodulating agents have been investigated to halt the disease progression including corticosteroids, IVIG, cyclosporin A, and anti-tumour necrosis factor-α (anti-TNF-α). They may be used singly or as combination therapy.

Systemic corticosteroids were one of the first recognised treatments for TEN. The theoretically increased risk of sepsis remains a concern especially in patients with extensive skin detachment. Most studies have found no increase in mortality with corticosteroid use (7). While some meta-analyses revealed no benefit in reducing mortality, two meta-analyses by Zimmermann et al. and Houschyar et al. showed that corticosteroids may improve survival (8–10). Three Japanese studies also demonstrated the beneficial effect of corticosteroids in reducing severe ocular sequelae (11–13). Although the beneficial effects of systemic corticosteroids were mostly based on results from retrospective and single-arm non-comparative studies, pulse corticosteroid therapy is recommended as one of the first-line treatments for SJS/TEN by Japanese treatment guidelines under appropriate infection control (14).

IVIG is another commonly used agent in the treatment for TEN. IVIG contains a broad range of naturally occurring autoantibodies and antibodies with anti-infectious activity, which are important in regulating immune functions. In particular, IVIG contains autoantibodies against a death receptor Fas. By interfering with the interaction between Fas and its ligand FasL, IVIG prevents apoptosis of keratinocytes. IVIG has been shown to be beneficial in terms of shorter lengths of hospital stay, fewer deaths, and faster healing time in retrospective studies in children (15–17). Small case series suggested that IVIG may reduce long-term keratopathy and subconjunctival fibrosis (18–21). The meta-analysis by Barron et al. found that increasing dose of IVIG was inversely correlated with mortality (22). However, other meta-analyses demonstrated no significant survival benefit of low-dose or high-dose IVIG in patients with TEN (15, 23). It is postulated that the inconsistent results might be attributable to the sensitivity of target cells to Fas, the concentration of IVIG used, and the relative proportions of agonistic and antagonistic anti-Fas autoantibodies in IVIG preparations (24).

Cyclosporin A is a calcineurin inhibitor. It inhibits the activation of CD4+ and CD8+ T cells, which subsequently inhibits the release of cytotoxic proteins such as perforin/granzyme B and granulysin. Retrospective studies and case series have demonstrated rapid re-epithelisation and a low mortality rate with cyclosporin A at an initial dose of 3–5 mg/kg/day (25–31). Several meta-analyses suggested a beneficial effect of cyclosporin A (7, 9, 10, 32–35). However, care should be taken in patients with pre-existing renal toxicity. Other possible adverse effects that were reported include posterior leucoencephalopathy, neutropenia, and nosocomial pneumonia (36).

Etanercept is a chimeric monoclonal antibody that targets TNF-α. It is shown that TNF-α, which upregulates FasL and acts a death receptor by itself, is overexpressed in affected keratinocytes of blistering lesions by immunohistochemistry of skin biopsies (37). Eliades et al. reviewed four paediatric patients ranging from 4 to 18 years old who had TEN with mucosal involvement of eyes, lips and oral cavity, and genitalia (38). They were treated with subcutaneous etanercept at a dose of either 0.8 mg/kg or 50 mg. Two patients were treated with a single dose, while the other two received a second dose in view of disease progression. The skin lesions responded to etanercept in 24–36 h with reduced erythema and halting of disease progression. The mean time to re-epithelialisation was 9 days. Six-month follow-up data were available and two patients were followed up by ophthalmologists for persistent dry eye disease. To date, there is only one randomised controlled trial by Wang et al. that compared etanercept and corticosteroids for the treatment for SJS/TEN in adult patients (39). The median time to re-epithelisation for the etanercept group was 14 days, which was shorter than the corticosteroid group. However, etanercept did not demonstrate significant mortality benefit. The overall incidence of adverse events was low including hypertension, hyperglycaemia, and gastrointestinal haemorrhage. It was recommended to consider a second dose in patients who continued to have fever or erythematous and/or oedematous skin lesions 36–48 h after the initial dose.

The combination of etanercept, corticosteroid, and cyclosporin A was reported in two paediatric patients for treatment for TEN. The first published case report by Gavigan et al. described an 11-year-old female with SJS/TEN triggered by sulfamethoxazole–trimethoprim (40). She was treated with IV methylprednisolone 30 mg/kg every 24 h on hospital days 1–5 and IV cyclosporin A 5 mg/kg every 24 h on hospital days 2–4. Cyclosporin A was discontinued after three doses as she developed seizures, which were possibly attributed to posterior reversible encephalopathy syndrome secondary to cyclosporin-induced hypertension. She received subcutaneous etanercept 25 mg on hospital days 4–5. Disease progression was halted on day 5 of hospitalisation. The total length of stay in the hospital and time to re-epithelialisation were not reported. Coulombe et al. reported a 17-year-old boy with carbamazepine-induced SJS who was treated with one dose of etanercept in combination with 3 days of dexamethasone and 7 days of cyclosporin A (41). He had rapid re-epithelialisation and was discharged within 14 days without clinical sequelae at 6 months. There have been some reports on the interaction between etanercept and cyclosporin, which highlight the importance of monitoring cyclosporin trough level to avoid significant underdosing or toxicity (42).

The combination of etanercept, corticosteroid, and IVIG was reported in three paediatric patients for treatment for TEN. Sibbald et al. reported a 2-year-old child who had ibuprofen/acetaminophen-induced SJS/TEN with a paediatric Severity-of-Illness Score for Toxic Epidermal Necrolysis (SCORTEN) of 4 (43). He was treated with prednisolone 1 mg/kg/day for 10 days, IVIG 1 g/kg/day for 4 days, and etanercept 0.4 mg/kg on days 6 and 8. His fever settled after initiation of etanercept, suggesting a possible direct role in halting disease. Holtz described a 9-year-old girl with phenobarbitone-induced SJS with vulvovaginal involvement (44). She developed transaminitis after receiving etanercept. Therefore, treatment was changed to a 3-day course of IVIG and IV steroids. Her vulvovaginal lesions showed significant improvement after 2 weeks. Zander et al. reported a 13-year-old boy who suffered from TEN due to trimethoprim–sulfamethoxazole (45). He was treated with IV methylprednisolone 5 mg/kg loading dose on day 2 of hospitalisation and maintenance therapy of 2.5 mg/kg IV every 8 h until day 5. He received subcutaneous etanercept 50 mg on day 2 and was given again on day 5. He received IVIG 1 g/kg every 24 h for the next 4 days. He was discharged after 13 days.

Our patient was, to date, the first paediatric case of TEN, which was successfully treated with a combination of four immunomodulatory agents, namely, corticosteroid, IVIG, cyclosporin A, and etanercept. He received IV pulse methylprednisolone 10 mg/kg/day for 3 days followed by gradual tapering in 1 week, IVIG 1 g/kg/day for 3 days, oral cyclosporin A 3 mg/kg/day on day 3 of hospitalisation (for a total of 3 weeks), and subcutaneous etanercept 0.8 mg/kg/dose on days 4 and 10. Since some studies have shown better ophthalmological outcomes and reduction in ocular sequelae with the use of etanercept, early consideration of etanercept in patients having TEN with significant ocular involvement is recommended. Our experience in this combination therapy is promising (46, 47). At the time of admission, the SCORTEN of our patient was 3 with a predicted mortality rate of 35%, and by day 5 of admission, the score decreased to 2 with a mortality rate of 12%. He had re-epithelisation after 13 days and he recovered well from amniotic membrane transplantation for the severe ocular involvement without clinical sequelae. He experienced no side effects from the multi-targeted therapy. Our case highlighted that prompt initiation of multi-targeted therapy was effective in halting disease progression in severe TEN. Its safety and good clinical outcome are also demonstrated. Long-term follow-up is warranted to detect late complications in him.

## Conclusion

Our patient illustrated successful treatment for trimethoprim–sulfamethoxazole-induced TEN with corticosteroid, IVIG, cyclosporin A, and etanercept. It highlighted that prompt initiation of multi-targeted therapy was effective in halting disease progression in severe TEN. This approach allows rapid tapering of corticosteroid to minimise complications of prolonged use of corticosteroid. It also demonstrated its safety and good clinical outcome.

## Data Availability

The original contributions presented in the study are included in the article/Supplementary Material, further inquiries can be directed to the corresponding author.
